# 9-(4-Nitro­phenyl­sulfon­yl)-9*H*-carbazole

**DOI:** 10.1107/S1600536811017818

**Published:** 2011-05-14

**Authors:** Nesimi Uludağ, Murat Ateş, Barış Tercan, Tuncer Hökelek

**Affiliations:** a Department of Chemistry, Faculty of Arts and Sciences, Namık Kemal University, 59030 Değirmenaltı, Tekirdağ, Turkey; b Department of Physics, Karabük University, 78050 Karabük, Turkey; c Department of Physics, Hacettepe University, 06800 Beytepe, Ankara, Turkey

## Abstract

In the title mol­ecule, C_18_H_12_N_2_O_4_S, the carbazole skeleton is nearly planar [maximum deviation = 0.037 (1) Å] and is oriented at a dihedral angle of 73.73 (5)° with respect to the benzene ring. An intra­molecular C—H⋯O hydrogen bond links a nitro O atom to the carbazole skeleton. In the crystal, inter­molecular C—H⋯O hydrogen bonds link the mol­ecules into a three-dimensional network. π–π contacts between inversion-related benzene rings [centroid–centroid distance = 3.7828 (8) Å] and two weak C—H⋯π inter­actions may also stabilize the structure.

## Related literature

For tetra­hydro­carbazole systems present in the framework of a number of indole-type alkaloids of biological inter­est, see: Saxton (1983 ▶). For related structures, see: Hökelek *et al.* (1994 ▶, 1998, ▶ 1999) ▶; Patır *et al.* (1997 ▶); Hökelek & Patır (1999 ▶). For the role of carbazole-based compounds in electroactive materials, see: Morin *et al.* (2004 ▶); Pasquali *et al.* (1993 ▶). For applications of chemically or electrochemically polymerized carbazole-based heterocyclic polymer systems, see: Sacak (1999 ▶); Santhanam & Sundaresan (1986 ▶).
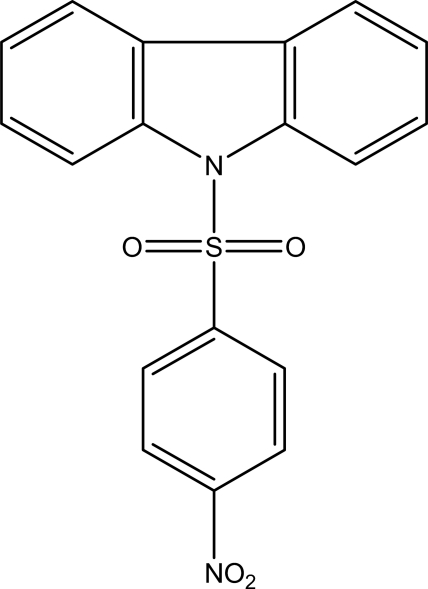


## Experimental

### 

#### Crystal data

C_18_H_12_N_2_O_4_S
*M* *_r_* = 352.37
Monoclinic, 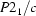

*a* = 7.4877 (2) Å
*b* = 11.7612 (3) Å
*c* = 17.3744 (4) Å
β = 90.119 (2)°
*V* = 1530.06 (7) Å^3^
*Z* = 4
Mo *K*α radiation
μ = 0.24 mm^−1^
*T* = 100 K
0.35 × 0.30 × 0.25 mm

#### Data collection

Bruker Kappa APEXII CCD area-detector diffractometer
Absorption correction: multi-scan (*SADABS*; Bruker, 2005 ▶) *T*_min_ = 0.921, *T*_max_ = 0.943
26862 measured reflections
3824 independent reflections
3269 reflections with *I* > 2σ(*I*)
*R*_int_ = 0.033

#### Refinement

*R*[*F* ^2^ > 2σ(*F* ^2^)] = 0.034
*wR*(*F* ^2^) = 0.091
*S* = 1.06
3824 reflections
226 parameters
H-atom parameters constrained
Δρ_max_ = 0.43 e Å^−3^
Δρ_min_ = −0.54 e Å^−3^

### 

Data collection: *APEX2* (Bruker, 2007 ▶); cell refinement: *SAINT* (Bruker, 2007 ▶); data reduction: *SAINT*; program(s) used to solve structure: *SHELXS97* (Sheldrick, 2008 ▶); program(s) used to refine structure: *SHELXL97* (Sheldrick, 2008 ▶); molecular graphics: *ORTEP-3 for Windows* (Farrugia, 1997 ▶); software used to prepare material for publication: *WinGX* (Farrugia, 1999 ▶) and *PLATON* (Spek, 2009 ▶).

## Supplementary Material

Crystal structure: contains datablocks I, global. DOI: 10.1107/S1600536811017818/su2273sup1.cif

Structure factors: contains datablocks I. DOI: 10.1107/S1600536811017818/su2273Isup2.hkl

Supplementary material file. DOI: 10.1107/S1600536811017818/su2273Isup3.cml

Additional supplementary materials:  crystallographic information; 3D view; checkCIF report

## Figures and Tables

**Table 1:** Hydrogen-bond geometry (Å, °) *Cg*2 and *Cg*3 are the centroids of the C1–C4/C4*A*/C9*A* and C5*A*/C5–C8/C8*A* rings, respectively.

*D*—H⋯*A*	*D*—H	H⋯*A*	*D*⋯*A*	*D*—H⋯*A*
C1—H1⋯O1	0.95	2.43	3.0210 (18)	121
C6—H6⋯O2^i^	0.95	2.57	3.4085 (19)	147
C14—H14⋯O2^ii^	0.95	2.46	3.2731 (18)	143
C1—H1⋯*Cg*3^iii^	0.95	2.94	3.7368 (15)	142
C12—H12⋯*Cg*2^iv^	0.95	2.71	3.4376 (15)	134

Symmetry codes: (i) 
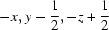
; (ii) 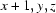; (iii) 
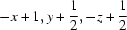
; (iv) 
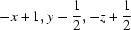
.

**Fig. 1.**
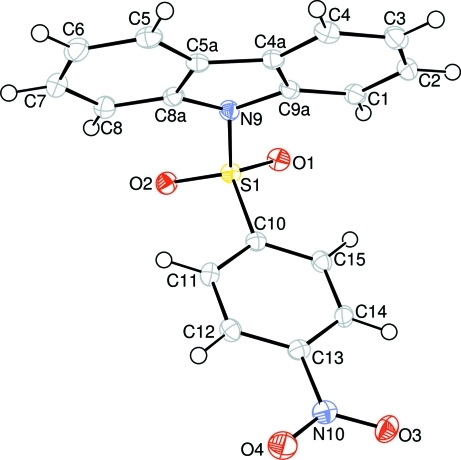
The molecular structure of the title molecule with the atom-numbering scheme. The displacement ellipsoids are drawn at the 50% probability level.

**Fig. 2.**
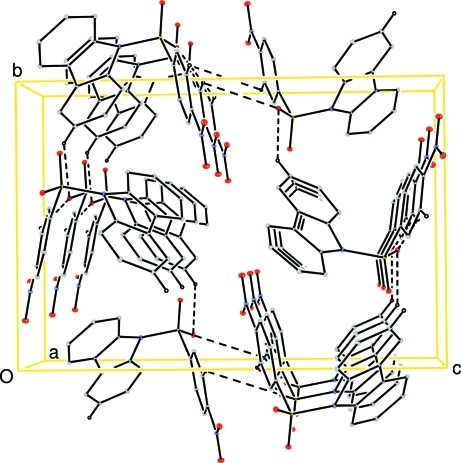
A view along the a-axis of the crystal packing of the title compound, showing the C-H···O interactions as dashed lines [H-atoms not involved in hydrogen bonding have been omitted for clarity; see Table 1 for details].

## supplementary crystallographic information

### Comment

Tetrahydrocarbazole systems are present in the framework of a number of indole-type alkaloids of biological interest (Saxton, 1983). The structures of tricyclic, tetracyclic and pentacyclic ring systems with dithiolane and other substituents of the tetrahydrocarbazole core, have been reported previously (Hökelek *et al.*, 1994; Patır *et al.*, 1997; Hökelek *et al.*, 1998; Hökelek *et al.*, 1999; Hökelek & Patır, 1999). Carbazole-based compounds play a very important role in electroactive materials (Morin *et al.*, 2004; Pasquali *et al.*, 1993). Carbazole-based heterocyclic polymer systems can be chemically or electrochemically polymerized to give products with a number of applications, such as rechargable batteries (Sacak, 1999) and electrochromic displays (Santhanam & Sundaresan, 1986). The title compound may be considered as a synthetic precursor of tetracyclic indole alkaloids of biological interests. The present study was undertaken to ascertain its crystal structure.

The title compound consists of a carbazole skeleton with a nitrophenylsulfonyl group (Fig. 1), where the bond lengths and angles are within normal ranges, and generally agree with those in the previously reported compounds mentioned above. In all structures atom N9 is substituted.

An examination of the deviations from the least-squares planes through individual rings shows that rings A (C1—C4/C4a/C9a), B (C4a/C5a/C8a/N9/C9a), C (C5a/C5—C8/C8a) and D (C10—C15) are planar. The carbazole skeleton, containing the rings A, B and C is also nearly coplanar [maximum deviation 0.037 (1) Å for atom C5] with dihedral angles of A/B = 1.10 (7), A/C = 1.94 (7) and B/C = 1.85 (7) °. The phenyl ring is oriented with respect to the carbazole skeleton at a dihedral angle of 73.73 (5)°. Atoms S1 and N10 are displaced by 0.095 (1) and 0.026 (1) Å, respectively, from the phenyl ring mean plane. The intramolecular C—H···O hydrogen bond (Table 1) links the nitro oxygen (O1) to the carbazole skeleton.

In the crystal intermolecular C—H···O hydrogen bonds link the molecules into a three dimensional network (Table 1 and Fig. 2). The π–π contacts between the phenyl rings, Cg4—Cg4^i^ [symmetry code: (i) 1 - x, 1 - y, -z, where Cg4 is the centroid of ring D (C10—C15)] may stabilize the structure, with a centroid-centroid distance of 3.7828 (8) Å, with perpendicular separation of 3.5543 (5) Å and a slipage of 1.295 Å. There also exist two weak C—H···π interactions involving rings A [Cg2 centroid of ring (C1—C4/C4a/C9a)] and C [Cg3 centroid of ring (C5a/C5—C8/C8a)], see Table 1.

### Experimental

For the preparation of the title compound, carbazole (3.0 g, 19.45 mmol) and 4-nitrobenzene-1-sulfonyl chloride (8.6 g, 38.91 mmol) were dissolved in dichloromethane (300 ml), and then tetramethyl ammonium hydrogen sulphate (0.3 g) and sodium hydroxide (40 ml, 50%) were added. The resulting mixture was stirred at room temperature for 24 h. It was then poured into water (200 ml) and dichloromethane (200 ml). The solvent was evaporated and the residue was purified by column chromatograpy using silica gel, and the product was crystallized from ethylacetate (yield; 4.2 g, 75.12%, m.p. 466 K).

### Refinement

H atoms were positioned geometrically with C—H = 0.95 Å for aromatic H atoms, and constrained to ride on their parent atoms, with *U*_iso_(H) = *1.2U*_eq_(C).

### Crystal data

**Table:**

C_18_H_12_N_2_O_4_S	*F*(000) = 728
*M**_r_* = 352.37	*D*_x_ = 1.530 Mg m^−^^3^
Monoclinic, *P*2_1_/*c*	Mo *K*α radiation, λ = 0.71073 Å
Hall symbol: -P 2ybc	Cell parameters from 8540 reflections
*a* = 7.4877 (2) Å	θ = 2.3–28.2°
*b* = 11.7612 (3) Å	µ = 0.24 mm^−^^1^
*c* = 17.3744 (4) Å	*T* = 100 K
β = 90.119 (2)°	Block, yellow
*V* = 1530.06 (7) Å^3^	0.35 × 0.30 × 0.25 mm
*Z* = 4	

### Data collection

**Table:**

Bruker Kappa APEXII CCD area-detector diffractometer	3824 independent reflections
Radiation source: fine-focus sealed tube	3269 reflections with *I* > 2σ(*I*)
graphite	*R*_int_ = 0.033
φ and ω scans	θ_max_ = 28.4°, θ_min_ = 2.1°
Absorption correction: multi-scan (*SADABS*; Bruker, 2005)	*h* = −9→10
*T*_min_ = 0.921, *T*_max_ = 0.943	*k* = −15→15
26862 measured reflections	*l* = −23→23

### Refinement

**Table:**

Refinement on *F*^2^	Primary atom site location: structure-invariant direct methods
Least-squares matrix: full	Secondary atom site location: difference Fourier map
*R*[*F*^2^ > 2σ(*F*^2^)] = 0.034	Hydrogen site location: inferred from neighbouring sites
*wR*(*F*^2^) = 0.091	H-atom parameters constrained
*S* = 1.06	*w* = 1/[σ^2^(*F*_o_^2^) + (0.0412*P*)^2^ + 0.8621*P*] where *P* = (*F*_o_^2^ + 2*F*_c_^2^)/3
3824 reflections	(Δ/σ)_max_ = 0.001
226 parameters	Δρ_max_ = 0.43 e Å^−^^3^
0 restraints	Δρ_min_ = −0.54 e Å^−^^3^

### Special details

**Table:**

Geometry. All esds (except the esd in the dihedral angle between two l.s. planes) are estimated using the full covariance matrix. The cell esds are taken into account individually in the estimation of esds in distances, angles and torsion angles; correlations between esds in cell parameters are only used when they are defined by crystal symmetry. An approximate (isotropic) treatment of cell esds is used for estimating esds involving l.s. planes.
Refinement. Refinement of F^2^ against ALL reflections. The weighted R-factor wR and goodness of fit S are based on F^2^, conventional R-factors R are based on F, with F set to zero for negative F^2^. The threshold expression of F^2^ > 2sigma(F^2^) is used only for calculating R-factors(gt) etc. and is not relevant to the choice of reflections for refinement. R-factors based on F^2^ are statistically about twice as large as those based on F, and R- factors based on ALL data will be even larger.

### Fractional atomic coordinates and isotropic or equivalent isotropic displacement parameters (Å^2^)

**Table:**

	*x*	*y*	*z*	*U*_iso_*/*U*_eq_	
S1	0.34852 (5)	0.62068 (3)	0.145676 (18)	0.01632 (10)	
O1	0.42494 (14)	0.73081 (8)	0.13727 (6)	0.0213 (2)	
O2	0.17638 (14)	0.59697 (9)	0.11410 (6)	0.0206 (2)	
O3	1.02664 (14)	0.30584 (10)	0.02810 (6)	0.0245 (2)	
O4	0.82092 (16)	0.17612 (9)	0.03061 (7)	0.0293 (3)	
C1	0.6420 (2)	0.67140 (12)	0.27841 (8)	0.0196 (3)	
H1	0.6511	0.7244	0.2373	0.023*	
C2	0.7764 (2)	0.66037 (13)	0.33332 (8)	0.0212 (3)	
H2	0.8799	0.7067	0.3293	0.025*	
C3	0.7637 (2)	0.58338 (13)	0.39410 (8)	0.0219 (3)	
H3	0.8579	0.5780	0.4306	0.026*	
C4	0.6144 (2)	0.51468 (12)	0.40160 (8)	0.0200 (3)	
H4	0.6050	0.4625	0.4431	0.024*	
C4A	0.47842 (19)	0.52351 (11)	0.34712 (8)	0.0169 (3)	
C5	0.2241 (2)	0.38241 (12)	0.38254 (8)	0.0207 (3)	
H5	0.2768	0.3555	0.4289	0.025*	
C5A	0.30977 (19)	0.46441 (11)	0.33801 (8)	0.0170 (3)	
C6	0.0606 (2)	0.34090 (13)	0.35799 (9)	0.0236 (3)	
H6	0.0017	0.2839	0.3872	0.028*	
C7	−0.0187 (2)	0.38190 (13)	0.29076 (9)	0.0242 (3)	
H7	−0.1305	0.3517	0.2749	0.029*	
C8	0.0618 (2)	0.46567 (13)	0.24653 (8)	0.0217 (3)	
H8	0.0060	0.4947	0.2015	0.026*	
C8A	0.22741 (19)	0.50524 (12)	0.27093 (8)	0.0171 (3)	
C9A	0.49421 (19)	0.60138 (12)	0.28645 (7)	0.0167 (3)	
N9	0.33693 (16)	0.59368 (10)	0.23925 (6)	0.0172 (2)	
N10	0.87222 (17)	0.27414 (11)	0.03883 (7)	0.0203 (3)	
C10	0.50149 (19)	0.51933 (11)	0.11037 (7)	0.0161 (3)	
C11	0.44419 (19)	0.40771 (12)	0.09937 (8)	0.0182 (3)	
H11	0.3229	0.3877	0.1079	0.022*	
C12	0.56592 (19)	0.32637 (12)	0.07590 (8)	0.0188 (3)	
H12	0.5308	0.2495	0.0685	0.023*	
C13	0.74049 (19)	0.36034 (12)	0.06344 (7)	0.0171 (3)	
C14	0.79846 (19)	0.47121 (12)	0.07233 (8)	0.0185 (3)	
H14	0.9189	0.4914	0.0619	0.022*	
C15	0.67725 (19)	0.55191 (12)	0.09676 (8)	0.0186 (3)	
H15	0.7133	0.6286	0.1042	0.022*	

### Atomic displacement parameters (Å^2^)

**Table:**

	*U*^11^	*U*^22^	*U*^33^	*U*^12^	*U*^13^	*U*^23^
S1	0.01736 (18)	0.01617 (17)	0.01543 (16)	0.00119 (12)	−0.00067 (12)	0.00134 (11)
O1	0.0250 (6)	0.0167 (5)	0.0222 (5)	0.0004 (4)	−0.0002 (4)	0.0026 (4)
O2	0.0177 (5)	0.0243 (5)	0.0199 (5)	0.0023 (4)	−0.0027 (4)	0.0017 (4)
O3	0.0162 (5)	0.0306 (6)	0.0267 (5)	0.0036 (4)	−0.0001 (4)	0.0016 (4)
O4	0.0294 (6)	0.0202 (6)	0.0384 (6)	0.0028 (5)	0.0022 (5)	−0.0033 (5)
C1	0.0222 (7)	0.0186 (7)	0.0180 (6)	−0.0013 (6)	0.0006 (5)	−0.0012 (5)
C2	0.0192 (7)	0.0214 (7)	0.0229 (7)	−0.0032 (6)	0.0002 (5)	−0.0050 (5)
C3	0.0215 (8)	0.0226 (7)	0.0216 (7)	0.0028 (6)	−0.0041 (5)	−0.0038 (5)
C4	0.0245 (8)	0.0178 (6)	0.0177 (6)	0.0033 (6)	−0.0018 (5)	−0.0007 (5)
C4A	0.0189 (7)	0.0146 (6)	0.0172 (6)	0.0015 (5)	0.0011 (5)	−0.0022 (5)
C5	0.0264 (8)	0.0171 (6)	0.0186 (6)	0.0022 (6)	0.0039 (5)	−0.0001 (5)
C5A	0.0194 (7)	0.0151 (6)	0.0166 (6)	0.0024 (5)	0.0017 (5)	−0.0031 (5)
C6	0.0256 (8)	0.0202 (7)	0.0250 (7)	−0.0036 (6)	0.0078 (6)	−0.0008 (6)
C7	0.0193 (8)	0.0275 (8)	0.0260 (7)	−0.0043 (6)	0.0036 (6)	−0.0034 (6)
C8	0.0191 (7)	0.0258 (7)	0.0201 (6)	−0.0008 (6)	0.0001 (5)	−0.0012 (5)
C8A	0.0182 (7)	0.0161 (6)	0.0171 (6)	0.0008 (5)	0.0032 (5)	−0.0010 (5)
C9A	0.0174 (7)	0.0174 (6)	0.0153 (6)	0.0021 (5)	−0.0014 (5)	−0.0031 (5)
N9	0.0175 (6)	0.0191 (6)	0.0149 (5)	−0.0014 (5)	−0.0011 (4)	0.0007 (4)
C10	0.0179 (7)	0.0174 (6)	0.0131 (6)	0.0013 (5)	−0.0012 (5)	0.0012 (5)
N10	0.0212 (7)	0.0222 (6)	0.0176 (5)	0.0034 (5)	−0.0014 (4)	0.0012 (4)
C11	0.0155 (7)	0.0195 (7)	0.0196 (6)	−0.0018 (5)	−0.0003 (5)	0.0026 (5)
C12	0.0209 (7)	0.0166 (6)	0.0189 (6)	−0.0020 (5)	−0.0010 (5)	0.0013 (5)
C13	0.0178 (7)	0.0197 (7)	0.0139 (6)	0.0032 (5)	−0.0008 (5)	0.0008 (5)
C14	0.0151 (7)	0.0238 (7)	0.0165 (6)	−0.0028 (5)	−0.0009 (5)	−0.0001 (5)
C15	0.0202 (7)	0.0181 (6)	0.0175 (6)	−0.0030 (5)	−0.0010 (5)	0.0010 (5)

### Geometric parameters (Å, °)

**Table:**

S1—O1	1.4237 (11)	C7—C8	1.388 (2)
S1—O2	1.4272 (11)	C7—H7	0.9500
S1—N9	1.6589 (11)	C8—H8	0.9500
S1—C10	1.7641 (14)	C8A—C8	1.390 (2)
O3—N10	1.2295 (17)	C8A—C5A	1.4023 (19)
O4—N10	1.2233 (17)	C9A—C1	1.387 (2)
C1—C2	1.391 (2)	C9A—C4A	1.4016 (19)
C1—H1	0.9500	N9—C8A	1.4349 (18)
C2—H2	0.9500	N9—C9A	1.4366 (17)
C3—C2	1.395 (2)	C10—C11	1.3941 (19)
C3—H3	0.9500	N10—C13	1.4783 (18)
C4—C3	1.385 (2)	C11—C12	1.383 (2)
C4—H4	0.9500	C11—H11	0.9500
C4A—C4	1.3929 (19)	C12—H12	0.9500
C4A—C5A	1.450 (2)	C13—C12	1.384 (2)
C5—C6	1.384 (2)	C13—C14	1.383 (2)
C5—H5	0.9500	C14—C15	1.381 (2)
C5A—C5	1.3938 (19)	C14—H14	0.9500
C6—C7	1.395 (2)	C15—C10	1.391 (2)
C6—H6	0.9500	C15—H15	0.9500
			
O1—S1—O2	120.07 (6)	C5A—C5—H5	120.6
O1—S1—N9	107.25 (6)	C6—C5—C5A	118.74 (13)
O1—S1—C10	108.53 (7)	C6—C5—H5	120.6
O2—S1—N9	106.87 (6)	C5—C5A—C4A	132.31 (13)
O2—S1—C10	108.71 (6)	C5—C5A—C8A	119.75 (13)
N9—S1—C10	104.29 (6)	C8A—C5A—C4A	107.94 (12)
C1—C9A—N9	129.31 (13)	C5—C6—C7	120.69 (14)
C1—C9A—C4A	122.17 (13)	C5—C6—H6	119.7
C4A—C9A—N9	108.53 (12)	C7—C6—H6	119.7
C8A—N9—S1	123.08 (9)	C8—C7—C6	121.65 (14)
C8A—N9—C9A	107.17 (11)	C8—C7—H7	119.2
C9A—N9—S1	120.19 (10)	C6—C7—H7	119.2
C11—C10—S1	118.95 (11)	C7—C8—C8A	117.18 (13)
C15—C10—S1	119.21 (11)	C7—C8—H8	121.4
C15—C10—C11	121.80 (13)	C8A—C8—H8	121.4
O3—N10—C13	117.65 (12)	C5A—C8A—N9	108.44 (12)
O4—N10—O3	124.28 (13)	C8—C8A—N9	129.49 (13)
O4—N10—C13	118.07 (12)	C8—C8A—C5A	121.96 (13)
C2—C1—H1	121.6	C10—C11—H11	120.4
C9A—C1—C2	116.86 (13)	C12—C11—C10	119.26 (13)
C9A—C1—H1	121.6	C12—C11—H11	120.4
C1—C2—C3	121.96 (14)	C11—C12—C13	118.00 (13)
C1—C2—H2	119.0	C11—C12—H12	121.0
C3—C2—H2	119.0	C13—C12—H12	121.0
C2—C3—H3	119.8	C12—C13—N10	118.56 (12)
C4—C3—C2	120.42 (13)	C14—C13—N10	118.00 (13)
C4—C3—H3	119.8	C14—C13—C12	123.44 (13)
C3—C4—C4A	118.77 (13)	C13—C14—H14	120.8
C3—C4—H4	120.6	C15—C14—C13	118.43 (13)
C4A—C4—H4	120.6	C15—C14—H14	120.8
C4—C4A—C5A	132.37 (13)	C10—C15—H15	120.5
C4—C4A—C9A	119.81 (13)	C14—C15—C10	119.05 (13)
C9A—C4A—C5A	107.82 (12)	C14—C15—H15	120.5
			
O1—S1—N9—C8A	163.45 (11)	N9—C8A—C8—C7	−176.89 (14)
O1—S1—N9—C9A	−54.69 (12)	C5A—C8A—C8—C7	−1.1 (2)
O2—S1—N9—C8A	33.47 (12)	S1—N9—C8A—C5A	149.32 (10)
O2—S1—N9—C9A	175.33 (10)	S1—N9—C8A—C8	−34.4 (2)
C10—S1—N9—C8A	−81.56 (12)	C9A—N9—C8A—C5A	3.29 (14)
C10—S1—N9—C9A	60.30 (11)	C9A—N9—C8A—C8	179.53 (14)
O1—S1—C10—C11	−168.31 (10)	S1—N9—C9A—C1	30.83 (19)
O1—S1—C10—C15	13.85 (12)	S1—N9—C9A—C4A	−149.61 (10)
O2—S1—C10—C11	−36.13 (12)	C8A—N9—C9A—C1	178.04 (14)
O2—S1—C10—C15	146.03 (11)	C8A—N9—C9A—C4A	−2.41 (14)
N9—S1—C10—C11	77.60 (11)	N9—C9A—C1—C2	−179.94 (13)
N9—S1—C10—C15	−100.24 (11)	C4A—C9A—C1—C2	0.6 (2)
C9A—C1—C2—C3	−0.5 (2)	C1—C9A—C4A—C4	−0.2 (2)
C4—C3—C2—C1	0.0 (2)	C1—C9A—C4A—C5A	−179.76 (12)
C4A—C4—C3—C2	0.4 (2)	N9—C9A—C4A—C4	−179.76 (12)
C5A—C4A—C4—C3	179.14 (14)	N9—C9A—C4A—C5A	0.65 (15)
C9A—C4A—C4—C3	−0.3 (2)	O3—N10—C13—C12	−179.94 (12)
C4—C4A—C5A—C5	3.1 (3)	O3—N10—C13—C14	0.47 (18)
C4—C4A—C5A—C8A	−178.12 (14)	O4—N10—C13—C12	−0.09 (18)
C9A—C4A—C5A—C5	−177.34 (14)	O4—N10—C13—C14	−179.68 (12)
C9A—C4A—C5A—C8A	1.40 (15)	S1—C10—C11—C12	−176.34 (10)
C5A—C5—C6—C7	−1.2 (2)	C15—C10—C11—C12	1.4 (2)
C4A—C5A—C5—C6	−179.66 (14)	C10—C11—C12—C13	−0.7 (2)
C8A—C5A—C5—C6	1.7 (2)	N10—C13—C12—C11	179.61 (12)
C5—C6—C7—C8	−0.5 (2)	C14—C13—C12—C11	−0.8 (2)
C6—C7—C8—C8A	1.6 (2)	N10—C13—C14—C15	−178.75 (12)
N9—C8A—C5A—C4A	−2.90 (15)	C12—C13—C14—C15	1.7 (2)
N9—C8A—C5A—C5	176.02 (12)	C13—C14—C15—C10	−0.94 (19)
C8—C8A—C5A—C4A	−179.48 (13)	C14—C15—C10—S1	177.20 (10)
C8—C8A—C5A—C5	−0.6 (2)	C14—C15—C10—C11	−0.6 (2)

### Hydrogen-bond geometry (Å, °)

**Table:**

Cg2 and Cg3 are the centroids of the C1–C4/C4A/C9A and C5A/C5–C8/C8A rings, respectively.

**Table:**

*D*—H···*A*	*D*—H	H···*A*	*D*···*A*	*D*—H···*A*
C1—H1···O1	0.95	2.43	3.0210 (18)	121
C6—H6···O2^i^	0.95	2.57	3.4085 (19)	147
C14—H14···O2^ii^	0.95	2.46	3.2731 (18)	143
C1—H1···Cg3^iii^	0.95	2.94	3.7368 (15)	142
C12—H12···Cg2^iv^	0.95	2.71	3.4376 (15)	134

Symmetry codes: (i) −*x*, *y*−1/2, −*z*+1/2; (ii) *x*+1, *y*, *z*; (iii) −*x*+1, *y*+1/2, −*z*+1/2; (iv) −*x*+1, *y*−1/2, −*z*+1/2.
